# Hazard Identification and Risk Prioritization Among Vendors and Visitors of a Traditional Wet Market in Padang, West Sumatra, Indonesia

**DOI:** 10.3390/ijerph23070941

**Published:** 2026-07-22

**Authors:** Aria Gusti, Wira Iqbal, Fitrahul Afifah

**Affiliations:** 1 Faculty of Public Health, Universitas Andalas, Padang 25163, West Sumatra, Indonesia; wiraiqbal@ph.unand.ac.id; 2 Faculty of Medicine, Universitas Negeri Padang, Bukittinggi 26136, West Sumatra, Indonesia; fitrahul.ipeh@gmail.com

**Keywords:** hazard identification, risk assessment, traditional wet market, occupational health and safety, vendors

## Abstract

**Highlights:**

**Public health relevance: How does this work relate to a public health issue?**

- Traditional wet markets in low- and middle-income countries remain under-regulated occupational health environments despite hosting millions of daily users.
- Nanggalo Market represents a common archetype of organically grown urban markets in Indonesia, where formal OHS systems are absent.

**Public health significance: Why is this work of significance to public health?**

- Seven of ten identified risks were classified as high-level, signaling an urgent need for structured hazard control in traditional market settings.
- The absence of fire suppression equipment and emergency evacuation routes creates catastrophic risk potential in densely occupied market spaces.

**Public health implications: What are the key implications or messages for practitioners, policy makers, and/or researchers in public health?**

- Local health authorities and market managers require a structured, activity-based risk matrix to prioritize control interventions in resource-limited settings.
- Activation of Occupational Health Posts at the primary health care level is recommended as a low-cost, community-based OHS mechanism.

**Abstract:**

Traditional wet markets are critical components of Indonesia’s urban food system, yet remain largely unexamined from an occupational health and safety (OHS) perspective. This study identified and prioritized OHS risks among vendors and visitors at Nanggalo Market, a high-density traditional market in Padang, West Sumatra. This was an exclusively qualitative, descriptive, non-probability (purposive) study; no actual occupational accidents, injuries, or exposure incidents were evaluated, and risk levels reflect participants’ perceptions together with researchers’ triangulated observations rather than objective incident records. An observational qualitative design combined elicitation surveys with 45 participants (20 vendors, 20 visitors, 5 market managers), direct observation, and in-depth interviews with 7 key informants. Risk assessment followed the AS/NZS 4360:2004 matrix, classifying risks by likelihood and consequence severity based on participant- and researcher-perceived evidence rather than recorded incident data. Ten potential hazards were identified across six activity zones: traffic and parking; buying and selling; culinary activities; building structures and floor conditions; security and stray animals; and emergency access. Seven risks were classified as high-level, including slipping on wet floors, lack of evacuation routes, lack of fire extinguishers, narrow circulation paths, poor toilet conditions, crowding, and traffic injuries, while three were moderate. All high-level risks lacked adequate controls. Recommended interventions span the full hierarchy of controls, prioritizing engineering and administrative measures, and propose community-based occupational health posts as a sustainable mechanism. These findings provide an evidence base for strengthening OHS governance in traditional wet markets across Indonesia and comparable low- and middle-income country settings.

## 1. Introduction

Traditional wet markets are an integral part of everyday life in urban and peri-urban areas of Southeast Asia, not only as places of commerce, but also as centers of life where food systems, social ties, and informal economic activities converge [1]. Indonesia alone has more than 14,000 registered traditional markets, hosting millions of vendors who have no other meaningful source of income and tens of millions of daily consumers; for the urban poor, these markets are often the only realistic source of affordable fresh food. However, their physical conditions frequently fall short of basic safety standards: overcrowded stalls, aging infrastructure, inadequate drainage, substandard electrical installations, and minimal emergency preparedness are widely documented features of traditional market environments, each representing a significant occupational health and safety (OHS) problem [2,3].

The emergence of COVID-19 has placed traditional wet markets in the global spotlight, with much attention focused on their potential role in the transmission of zoonotic diseases [4,5]. The resulting policy discussions narrowed public health attention toward infectious disease risks, leaving the physical and structural OHS hazards that injure and impair vendors and visitors, independent of any pathogen, comparatively understudied, particularly in low- and middle-income countries where regulatory intent and on-the-ground practice diverge most sharply [6,7]. In practice, workers in informal market environments face an extensive occupational risk profile: musculoskeletal strain from prolonged standing and heavy lifting, thermal stress from poor ventilation, chemical exposure from cleaning agents, traffic hazards at crowded entrances, slip-and-fall risk from wet floors, and fire hazards from open-flame cooking near combustible materials. Yet, few of these hazards have been systematically documented, let alone addressed through structured control programs.

Governance of Indonesia’s traditional markets remains fragmented. Although a formal legal framework exists (Law No. 1 of 1970 concerning Occupational Safety and Government Regulation No. 50 of 2012 on the Occupational Health and Safety Management System, containing explicit mandates for workplace hazard management), these instruments were written for formal employment relationships [8] and do not readily extend to the self-employed, informal traders who dominate traditional markets and lack employment contracts, social security registration, or access to occupational health services. Where market management units exist, they typically prioritize revenue collection over safety oversight, producing a systematic lack of OHS accountability in some of the country’s most densely populated and physically hazardous work environments. This pattern is not unique to Indonesia: studies from African market contexts document remarkably similar conditions of ergonomic, chemical, fire, and traffic hazards under comparable informality and regulatory exclusion [9,10].

Nanggalo Market, located in the densely populated Nanggalo sub-district of Padang, West Sumatra, is a prime example of this pattern: it did not emerge from any formal planning process, but has grown organically since the 1960s, with infrastructure investment and spatial organization lagging far behind commercial and population growth (site characteristics are detailed in Section 2.1). Previous research at the site has revealed significant issues in environmental sanitation, inadequate waste management, unreliable water supplies, and food-handling practices that fall far short of acceptable standards [11]. What has been missing until now is a systematic effort to comprehensively map the market’s OHS risk landscape: a multi-zone hazard identification exercise that goes beyond sanitation, encompasses the breadth of physical and structural risks present across the market’s various activity zones, and, crucially, captures how these risks are actually perceived and experienced by vendors, visitors, and market managers.

Bridging this gap requires an approach that can capture the full diversity of hazards across a market’s spatial zones while remaining practical within typical LMIC research constraints. The AS/NZS 4360:2004 risk management standard ranks the severity of consequences against the likelihood of occurrence to guide control priorities [12]. Its systematic application to traditional wet markets in Indonesia has not been documented previously.

Existing OHS literature on traditional markets remains narrow in scope: most studies examine a single hazard category, such as fire risk or food contamination, or focus on formal retail environments with fundamentally different infrastructure and enforcement contexts than unplanned wet markets [13,14,15]. A comprehensive, multi-hazard assessment that considers the full activity profile of Indonesia’s traditional wet markets and systematically incorporates the perspectives of traders, visitors, and management personnel has not been published previously.

This study was designed to address this gap. Conducted at Nanggalo Market, it presents a multi-zone hazard identification and risk prioritization exercise combining a structured elicitation survey, systematic direct observation, and triangulated key-informant interviews. It makes three main contributions: (i) a multi-stakeholder elicitation approach capturing differing hazard perceptions across key actor groups; (ii) systematic application of the AS/NZS 4360:2004 risk matrix to an Indonesian wet market context; and (iii) an activity-based risk control matrix translating identified hazards into prioritized, actionable recommendations spanning the full control hierarchy. These findings are intended as a practical reference for local health authorities, market management units, and policymakers involved in traditional market governance in Indonesia and comparable low- and middle-income settings.

## 2. Materials and Methods

### 2.1. Study Design and Setting

This study employed a qualitative, exploratory-descriptive observational design. The design was chosen to generate a comprehensive, context-specific inventory of occupational health and safety (OHS) hazards at a single, purposively selected market site, rather than to produce statistically representative estimates of hazard prevalence for a defined population. The design triangulated three complementary, non-probability-based methods: (1) a structured elicitation survey administered to a purposively selected sample of vendors, visitors, and market management personnel; (2) systematic direct observation using structured checklists; and (3) in-depth interviews with purposively selected key informants. This multi-method, multi-stakeholder approach is consistent with rapid hazard-identification designs commonly used for occupational risk mapping in informal work settings, where the primary aim is descriptive and prioritization-oriented rather than explanatory or inferential. The study was conducted at Nanggalo Market, Nanggalo District, Padang City, West Sumatra, Indonesia. The market occupies approximately 217,250 m^2^, accommodates approximately 170 permanent kiosk vendors and 350 street vendors, and attracts thousands of visitors daily. Nanggalo Market has been operating since the 1960s and has gradually expanded over time, without ever being guided by spatial planning or a formal, structured occupational safety and health system.

### 2.2. Participants and Sampling Strategy

Participants were recruited using purposive (non-probability) sampling. This strategy was chosen because the study’s objective was to elicit and triangulate the breadth of hazards known to different stakeholder groups across six pre-defined activity zones, a task that requires participants with direct, first-hand exposure to each zone rather than a sample drawn to be statistically representative of the entire vendor, visitor, or management population of Nanggalo Market.

Sampling frame and recruitment procedure: The sampling frame comprised all vendors, visitors, and market management personnel present at Nanggalo Market during the data collection period. Research team members approached prospective participants directly at their kiosks or stalls (vendors) or while shopping within the market (visitors), across all six activity zones and on both weekday and weekend/peak-traffic days, to capture the full range of spatial and temporal hazard exposure. Recruitment continued within each stakeholder group until the pre-specified quota (20 vendors, 20 visitors, 5 market management personnel) was reached.

Inclusion criteria: (a) Vendors currently operating a permanent kiosk or itinerant stall at Nanggalo Market for at least 1 year; physically present at the site during the data collection period; aged ≥18 years; able to communicate in Bahasa Indonesia; and willing to provide verbal informed consent. (b) Visitors aged ≥18 years; present at the market at the time of data collection; visiting the market at least weekly to ensure sufficient familiarity with its physical conditions to meaningfully report hazards; and willing to provide verbal informed consent. (c) Market management personnel holding an active administrative or supervisory position within the Market Management Unit at the time of data collection.

Exclusion criteria: individuals aged <18 years; visitors judged to have insufficient familiarity with the market’s layout and conditions to reliably report hazards (e.g., first-time visitors); individuals unable to communicate verbally due to health or language barriers; and any prospective participant who declined consent.

Hazard identification was conducted through elicitation with 45 purposively selected participants: 20 vendors, 20 visitors, and 5 market management personnel. This sample size was set a priori, informed by comparable multi-stakeholder hazard-elicitation studies conducted in traditional market settings, and judged to provide adequate coverage given the concrete, bounded nature of the elicitation task (identifying hazards within six pre-defined activity zones) and the limited number of management personnel available at the site. For context, the vendor population at Nanggalo Market comprises approximately 170 permanent kiosk vendors and 350 itinerant street vendors (~520 vendors in total), while the daily visitor population fluctuates and is not formally enumerated; the Market Management Unit comprises a small number of staff, of whom 5 participated in elicitation, and 1 (the unit head) participated as a key informant. Because participants were selected via purposive, non-probability sampling rather than random or systematic sampling from a defined sampling frame, finite-population probability-based sample-size formulas (e.g., the Slovin or Cochran formulas) are not statistically appropriate for this design and were therefore not applied; such formulas presuppose random selection from an enumerated population and a target margin of error for population-level inference, neither of which corresponds to the descriptive, non-probabilistic aim of this study. The sample size was instead justified qualitatively, on the criteria described above. Elicitation participants were asked to freely identify hazards they had encountered in each of the six pre-defined activity zones. Risk assessment triangulation was performed with 7 key informants (3 vendors, 3 visitors, and 1 head of the Market Management Unit), selected from among the 45 elicitation participants using criterion (purposive) sampling based on (i) length of experience at the market (≥5 years) and (ii) willingness to participate in a longer in-depth interview; the head of the Market Management Unit was included by virtue of holding that position. All participants provided verbal informed consent prior to data collection.

Because sampling was purposive and non-probabilistic, this sample should not be regarded as statistically representative of the full population of vendors, visitors, or management personnel at Nanggalo Market. The elicitation frequencies reported in Section 3 (e.g., the proportion of respondents endorsing a given hazard) describe the relative salience of hazards within this specific sample and are used to rank and prioritize hazards for the subsequent risk matrix; they should not be interpreted as population-level prevalence estimates. The implications of this non-probability design for the generalizability of the findings are discussed further in Section 4 and in the Limitations (Section 4, final paragraph).

### 2.3. Data Collection

Data collection proceeded in three stages. First, structured observation checklists were used to document physical conditions across all six activity zones: (1) traffic and parking area, (2) buying and selling activities, (3) culinary activities, (4) building structure, vendor tables, and floor conditions, (5) security and stray animals, and (6) emergency access. Second, elicitation surveys were administered individually to the 45 participants, who were asked to identify hazards in each zone without prompting. Third, in-depth interviews with the 7 key informants were conducted to triangulate and validate the identified hazards and current control measures. Interviews were conducted in Bahasa Indonesia and audio-recorded with participant consent. The structured observation checklist and the elicitation and interview guides were developed by the research team based on the six activity zones identified during preliminary site visits and on the AS/NZS 4360:2004 risk management framework, and were reviewed internally by the research team for content coverage prior to use. However, these instruments were not formally validated (e.g., through an expert-panel content validity procedure) nor pilot-tested on a separate sample prior to the main data collection. This is acknowledged as a limitation of the study (Section 4).

### 2.4. Risk Assessment Method

Risk assessment followed the AS/NZS 4360:2004 standard. Each identified potential hazard was assessed by its associated risk, then evaluated on two dimensions: Consequence (C) and Likelihood/Probability (L). The Consequence scale ranged from 1 to 5 (1 = Negligible: no injury, negligible impact; 2 = Minor: first-aid-level injury or minor property/operational impact; 3 = Moderate: injury requiring medical treatment or moderate disruption; 4 = Major: serious injury or major disruption; 5 = Catastrophic: death or multiple serious injuries, or catastrophic disruption). The Likelihood scale ranged from A to E (A = Rare: may occur only in exceptional circumstances; B = Unlikely: could occur at some time; C = Possible: might occur at some time; D = Likely: will probably occur in most circumstances; E = Almost certain: expected to occur in most circumstances), consistent with standard AS/NZS 4360:2004 descriptors. The risk level was determined by the intersection of these scores on the standard risk matrix. Risk levels were categorized as Low, Moderate, High, or Extreme. Consequence and Likelihood scores for each hazard were assigned by the research team based on triangulated evidence from direct observation, elicitation frequency, and key-informant interviews. Scores were discussed jointly by the assessors until consensus was reached; in the few instances of initial disagreement, the assessors re-examined the underlying observation and interview data together and agreed on a final score by discussion. A formal quantitative inter-rater agreement statistic (e.g., Cohen’s kappa or percentage agreement) was not calculated, since scoring was conducted through group consensus rather than fully independent parallel scoring. This reliance on team consensus rather than independently verified inter-rater agreement is acknowledged as a limitation (Section 4). For each identified high or extreme risk, current controls were documented, and recommendations were formulated in accordance with the ISO 45001:2018 hierarchy of controls [16].

### 2.5. Data Analysis

Elicitation data were analyzed using frequency tabulation to characterize the relative salience of hazards reported by respondents within each of the six activity zones (reported in Section 3.2). Hazard identification for the risk assessment (Table 1 and Table 2) was based on triangulation of elicitation reports, direct observation, and key-informant interviews within each zone, not on elicitation frequency alone; all ten hazards identified through this triangulation process were carried forward to formal AS/NZS 4360:2004 risk assessment (Section 2.4), regardless of their elicitation frequency. Frequency ranking (Section 3.2) is therefore reported as a separate, descriptive characterization of how commonly each hazard was recognized by respondents, and was not used as an inclusion or exclusion criterion for the risk matrix. This distinction is important because frequency of endorsement and risk severity are conceptually distinct and need not coincide: for example, the absence of portable fire extinguishers was endorsed by only 37.8% of respondents, yet was classified as a high risk owing to its consequence severity, illustrating that the risk matrix in Table 2 captures severity-based prioritization independent of raw reporting frequency. Qualitative data from interviews were analyzed using the Miles and Huberman thematic framework: data reduction, data display, and conclusion drawing/verification [17]. Findings from observation, elicitation, and interviews were triangulated to confirm hazard identification and assess the adequacy of existing controls. Consistent with the study’s exploratory-descriptive qualitative design (Section 2.1), all quantitative summaries reported in this manuscript (e.g., elicitation frequencies and percentages in Section 3.2) are descriptive only; no inferential statistical tests (e.g., significance testing or regression modeling) were performed, and none are appropriate given the non-probability sampling strategy used. The study and its findings should accordingly be read as a qualitative, descriptive risk-mapping exercise rather than as a statistically inferential study.

### 2.6. Ethical Considerations

This study did not involve any biological intervention on human subjects. All human participation was limited to information provision through surveys and interviews. Prior to participation, all participants received clear and comprehensive information regarding the study objectives, procedures, potential risks, and anticipated benefits.

Informed consent was obtained from all participants before data collection commenced. Participants were assured that all collected data would be treated with strict confidentiality and anonymity. Personal identifiers were removed from all records, and data were securely stored and accessible only to the research team for academic purposes. Participants were explicitly informed of their right to decline participation or to withdraw at any stage of the study without academic, personal, or professional consequences. No coercion or undue influence was exercised during recruitment or data collection.

The study presented minimal risk to participants. Throughout the research process, all reasonable measures were taken to protect participants’ dignity, autonomy, rights, and well-being.

## 3. Results

### 3.1. Hazard Identification by Activity Zone

A systematic hazard identification across the six activity zones of Nanggalo Market identified 10 key potential hazards, each associated with specific risks to the safety and health of vendors, visitors, and market management staff. These hazards were distributed across all six zones rather than concentrated in a single area or activity type (interpreted further in Section 4).

Zone 1, which encompasses the traffic and parking area at the market entrance, presents two primary hazards: high traffic density on weekends and holidays and the absence of directional signage at vehicle entry and exit points. Both of these hazards stem directly from the lack of traffic management planning, where the volume of vehicles accessing the market on peak days far exceeds the capacity of available road space and parking areas. Zone 2, which encompasses the trading activities on the main market floor, presents two additional hazards: narrow circulation paths between vendor stalls and the absence of a vendor zoning system. These two conditions interact to create a disorganized spatial environment where pedestrian flow is impeded, and visitor navigation is inefficient, simultaneously increasing both physical and safety risks. Zone 3, the culinary activity zone, presents one primary hazard: uncovered food displayed in open stalls, with direct implications for food safety and the potential for foodborne disease transmission through vector contact and contamination of the surrounding environment.

Zone 4, which encompasses building structure, merchandise counters, and floor conditions, had the highest number of hazards among the study’s zones, reflecting the multiple impacts of aging infrastructure, inadequate maintenance, and wet conditions resulting from fresh fish and meat handling. The two primary hazards identified for this zone were slippery, wet floors in the fish and meat areas and dark, inadequate restroom facilities, both of which represented the highest frequency and most structurally entrenched risks in the overall study. Zone 5, which encompasses security conditions and stray animal activity, identified the free movement of cats and goats throughout the market as its primary hazard, with consequences including contamination of merchandise, food safety issues, and an increased risk of zoonotic disease transmission. Zone 6, the emergency access zone, generated the two most critical hazards in the overall study: the absence of evacuation route signs in the market area and the lack of portable fire extinguishers throughout the market.

Table 1 presents a consolidated summary of the ten primary potential hazards and their associated risk outcomes by activity zone. Complete elicitation frequency data, including all hazard items identified per zone and endorsement counts disaggregated by respondent group, are provided in Table S1 of the Supplementary Material.

### 3.2. Hazard Elicitation Frequencies by Zone

**Figure 1:**
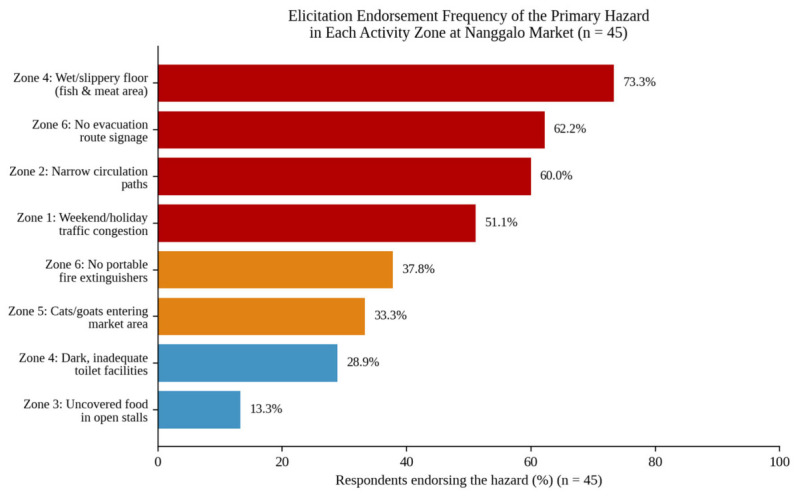
Elicitation endorsement frequency (%) of the primary hazard identified in each activity zone at Nanggalo Market (*n* = 45). Bars are shaded to show endorsement rate (dark red ≥50%; orange 30–49%; blue <30%); shading indicates reporting frequency only, not risk-matrix severity (see Table 2).

Analysis of information collection frequency data across six activity zones revealed striking variations in both the volume and types of hazards reported by respondent groups, reflecting differences in how traders, visitors, and market managers experience and understand risks within the same physical environment. This analysis is shown in Figure 1.

Zone 4, which encompasses the building structure, merchandise counters, and floor conditions, recorded the highest number of hazard reports across the entire study, with respondents identifying 10 distinct hazards in this zone. The concentration of so many hazard categories in this single zone reflects the cumulative impact of neglected facility maintenance and the absence of a structured physical management system at Nanggalo Market. Of all hazard items listed across all zones, the slippery and wet floors of the fish and meat stalls had the highest frequency of endorsement, with 33 of 45 respondents (73.3%) identifying them as a significant hazard. This finding was consistent across all three respondent groups (vendors, visitors, and managers). Additionally, minor injuries from cutting and processing food in the same zone were reported by 10 respondents, indicating an additional occupational health burden alongside the risk of slipping in the same area. Meanwhile, 13 respondents (28.9%) reported dark and inadequate toilet conditions, reflecting a dual concern: the risk of injury from poor lighting and the sanitation implications of facilities that are far from adequate in number and condition to serve the daily volume of visitors.

Zone 6, which covers emergency access, recorded the second-highest concentration of high-frequency reports, despite containing only two hazard items, underscoring the critical nature of the identified deficiencies. The absence of evacuation route signs was reported by 28 of the 45 respondents (62.2%), making it the hazard item with the second-highest frequency of endorsement in the entire study. Seventeen respondents (37.8%) identified the absence of portable fire extinguishers. Both items were completely absent from the market, and their absence was acknowledged by nearly two-thirds of all respondents combined.

In Zone 2, which encompasses buying and selling activities, 27 respondents (60.0%) identified narrow circulation paths between vendor stalls, making it the third-most-frequently reported hazard overall; endorsement was highest among visitors (16 of 20 respondents).

Zone 1, which includes traffic and parking areas, was the fourth most frequently reported source of hazard, with 23 respondents (51.1%) citing traffic congestion on weekends and holidays as a significant problem, and 6 respondents additionally reporting the absence of directional signage at market entrances and exits.

In Zone 5, which covers security and stray animals, 15 respondents (33.3%) reported that cats and goats freely enter the market area, posing a significant hazard; the most frequently cited consequence is disruption to merchandise. Zone 3, which covers culinary activities, recorded the lowest total number of reports of all zones, with uncovered food identified by 6 respondents (13.3%) as a primary concern.

Overall, the frequency data revealed a hazard landscape concentrated on the market’s physical infrastructure, particularly floor conditions and emergency preparedness systems, with secondary concerns in spatial management and traffic safety. Reporting patterns differed by respondent group: vendors reported hazards encountered in their daily work routines more frequently, visitors reported hazards related to comfort and safety while shopping more frequently, and market managers reported structural and emergency-access issues more frequently.

### 3.3. Risk Assessment and Prioritization

Table 2 presents the full risk assessment matrix, including consequence score (K), likelihood score (P), composite risk level, current controls, and recommended interventions for all ten identified risks. Seven risks were classified as high-level and three as moderate. As detailed in Section 2.4, these classifications reflect consensus-based scoring by the research team, drawing on triangulated observation, elicitation, and interview evidence, rather than independent parallel assessment or objective incident/exposure data; the risk levels reported below should therefore be interpreted as informed relative rankings rather than statistically validated measurements.

### 3.4. Current Control Adequacy

An assessment of existing control measures at Nanggalo Market revealed a gap between the severity of the identified risks and the adequacy of the responses implemented. Of the seven risks classified as high, only one, wet and slippery floors in the fish and meat stalls, had a documented control measure, consisting of daily floor mopping by vendors and verbal warnings to visitors. No structural controls (e.g., non-slip flooring, anti-slip coating, corrected floor slope, or covered drainage) were observed for this hazard. For the remaining six high-risk hazards, no control measures were identified through either direct observation or informant testimony: (i) no portable fire extinguishers were present in any market zone; (ii) no evacuation route signage was installed anywhere in the market; (iii) narrow circulation routes, reported by 60% of respondents, showed no evidence of kiosk reconfiguration, a one-way traffic system, or enforcement of stall-boundary rules; (iv) weekend/holiday traffic congestion at the market entrance was not managed by the Transportation Agency, and no parking or directional signage had been installed; (v) no vendor zoning system was in place, and no documented improvement plan was identified; and (vi) the dark, inadequate restroom facilities had received no lighting or facility upgrades. No risk register, inspection schedule, or designated safety officer was identified at the site.

Of the three moderate risks, partial control was found in only one case. Parking congestion on weekends and holidays was managed by informal parking attendants using only hand signals, with no floor markings, physical barriers, formal signage, or documented procedures, and no backup coverage when attendants were absent. For the remaining two moderate risks (foodborne illness from open food service and merchandise contamination from stray animals), no documented control measures were identified: no standardized food covers, no animal control schedule, and no related procedures were found at the time of data collection.

In total, only two documented control measures were identified across all ten assessed hazards: daily floor mopping in Zone 4, and informally organized parking assistance in Zone 1. No documented risk register, regular inspection schedule, or formally designated safety officer was identified at Nanggalo Market during the data-collection period. The interpretive implications of this pattern of control adequacy are addressed in Section 4 (Discussion).

## 4. Discussion

To our knowledge, this is among the first studies to conduct a comprehensive OHS risk assessment of a traditional wet market in West Sumatra, mapping 10 hazards across six activity zones, seven of which are categorized as high risk. These classifications rest on consensus-based scoring by the research team rather than independent assessment or objective outcome data (Section 2.4), and should be read as informed relative rankings rather than validated risk measurements. The range of hazards identified, from physical injury to sanitation and fire risks to the lack of emergency access, illustrates the complexity of OHS governance in markets that have grown naturally without adequate safety planning. What makes this situation difficult to address is not simply budgetary or human resource constraints, but a more fundamental governance gap: traditional markets in Indonesia are owned and managed by the government, but the vendors who staff them are informal workers legally excluded from national labor protections [18]. They trade daily in an environment fraught with multiple hazards, yet no one is officially responsible for their safety. This structural ambiguity has allowed hazards to accumulate for decades without ever triggering systematic corrective action, and the study’s findings clearly reflect the consequences of prolonged neglect.

The near-total absence of documented controls (Section 3.4) suggests that the gap observed at Nanggalo Market reflects systemic institutional neglect rather than an incidental or purely resource-driven shortfall. Because market management units in this setting typically prioritize revenue collection over safety oversight, hazard control appears to occur reactively, if at all, rather than through a planned inspection or reporting cycle. This pattern is consistent with governance patterns documented in other low- and middle-income informal market settings, discussed further below.

The prevalence of high risk at Nanggalo Market is not surprising when viewed in a broader context. Systematic reviews of OHS conditions in Sub-Saharan African markets show a nearly identical pattern: most occupational hazards are uncontrolled or only minimally managed, largely due to the informal status of traders and the absence of effective regulatory oversight [9]. Similar concerns have been documented across South and Southeast Asian contexts, where market workers report daily exposure to ergonomic stress, excessive heat, chemicals from detergents and pesticides, and the risk of traffic accidents, all without access to meaningful formal OHS protection. The findings of this study extend this line of evidence to the Indonesian context, and the message is clear: OHS control gaps in traditional markets are not local issues stemming from particular cultural or geographic idiosyncrasies, but rather reflect the structural nature of informal workplaces in developing countries, where occupational safety regulations do exist in the form of written rules but are rarely actually present on the market floor where the hazards are most acutely felt [19,20]. Bridging the gap between what is written in policy and what happens on the ground cannot be resolved simply by revising regulations; it requires concrete capacity-building at the market-management level, particularly for management units that currently lack the technical knowledge or institutional mandate to implement systematic hazard control.

The fact that wet, slippery floors in the fish and meat stalls emerged as the most frequently endorsed hazard is consistent with prior evidence indicating that slips and falls are a leading cause of injury in wet market environments [13,14]. Standing water, blood, fish scales, and organic debris create a near-frictionless surface, posing particular risk to vendors who stand for extended periods while lifting heavy loads. That the only identified control was daily mopping and verbal warnings, an administrative approach entirely dependent on human compliance, is itself telling: ISO 45001:2018 indicates that engineering controls, such as non-slip flooring, textured wet-work surfaces, corrected floor slopes, and drainage to prevent pooling, are inherently more effective because they reduce hazard exposure independent of behavioral compliance [16,21].

The absence of portable fire extinguishers and evacuation route signage throughout Nanggalo Market is among the most concerning findings of this study, given that both deficiencies co-occur. Fires in Indonesian traditional markets, including several in West Sumatra, are a recurring and well-documented hazard, typically involving densely packed buildings, flammable materials stored near cooking areas, and outdated electrical installations [22]. This hazard’s high-risk classification, at the border of the Extreme threshold, reflects the market’s physical density and the proximity of open-flame cooking to dry-goods stalls. The culinary zone is particularly vulnerable, with open-flame stoves, under-counter LPG cylinders, and poorly ventilated cooking areas positioned in close proximity. Providing portable fire extinguishers across all market zones and installing visible evacuation signage represent minimum engineering and administrative control measures consistent with the ISO 45001:2018 hierarchy of controls, and are prioritized accordingly in Table 2.

The traffic-related hazard identified in Zone 1, heavy weekend/holiday congestion at the market entrance, is consistent with the broader traffic-safety context of Padang and West Sumatra. Official data from the West Sumatra Regional Police Traffic Directorate (Polda Sumbar) recorded 3500 traffic accidents province-wide in 2022, rising to 3700 in 2023, with victims and at-fault parties concentrated among the 16–30-year productive age group [23]. Municipal statistics from Statistics Indonesia (BPS) for Padang City have similarly recorded several hundred traffic accidents annually in recent years, with associated fatalities and severe injuries [24]. These figures corroborate that traffic-related injury is a substantiated public safety concern in the study area and provide external context for participants’ reported concerns about congestion-related accident risk at the market entrance. We emphasize, however, that these statistics describe city- and province-wide traffic accidents in general and are not disaggregated to the immediate vicinity of Nanggalo Market specifically; no site-specific traffic accident or near-miss records for the market entrance were available to this study, and this remains a limitation (Section 4).

The multi-stakeholder elicitation approach yielded a richer hazard map than a single-group survey would have produced. Visitors more often mentioned narrow circulation paths and stray-animal disturbances, likely because they lack the vendors’ familiar spatial knowledge of the market. Vendors more often reported slippery floors and cutting injuries in the fish and meat area, hazards most acutely felt by those standing on these surfaces for extended daily periods. Market managers showed greater awareness of structural weaknesses and emergency-access gaps than the other two groups, yet this awareness had not translated into corrective action. These divergent perceptions have direct implications for OHS program design: relying on any single stakeholder group risks underestimating hazards that are more visible to, or more acutely experienced by, the other groups [25,26]. The elicitation framework used here, which engaged all three groups without prior direction, offers a replicable model for hazard identification in comparable traditional markets.

The risk of foodborne illness from uncovered food in the culinary zone was classified as moderate in this study’s matrix, reflecting a lower consequence score than acute physical injury; however, this classification should not be read as indicating low risk in practice. Traditional wet markets that handle fresh meat, fish, and cooked food in close proximity without physical separation create conditions conducive to enteric pathogen transmission [4,22]. Respondents described contamination pathways as routine and normalized: flies moving between raw fish, rice packets, and open food; cockroaches and rats near storage and serving areas; and raw and ready-to-eat foods sharing surfaces without barriers. Studies of Indonesian markets consistently find that food hygiene practices remain far below safe thresholds, not because vendors are unconcerned but because they lack basic tools to maintain them (adequate food coverings, refrigeration, vector-protected storage) [27,28]. Findings from Gusti et al.’s study of preventive health behaviors among farmers in West Sumatra reinforce that knowledge and social support do not translate into protective behavior when the physical work environment does not support it [29]; the WHO has similarly emphasized that hygiene education alone is insufficient without structural interventions that change the physical conditions of food sale [27]. OHS governance in environments like Nanggalo Market must therefore operate on two levels, improving infrastructure as a prerequisite and building behavioral capacity as a follow-up, since prioritizing the latter without the former is unlikely to produce lasting change. The moderate risk classification in this study should accordingly be understood as a starting point rather than a conclusion, given the market’s high daily visitor volume and the chronic nature of exposure.

The presence of cats and goats roaming freely in market areas carries impacts that extend beyond messy merchandise or food contaminated with animal fur. Animals moving freely between fish stalls, meat counters, and prepared food preparation areas carry fecal contamination, creating a direct pathway for the transmission of zoonotic pathogens to food surfaces and human hands. In the post-COVID-19 context, where the international public health community has increased scrutiny of biosafety conditions in traditional wet markets globally, these findings have relevance beyond the boundaries of a single market or city [4,5,30]. While the zoonotic risk profile of domesticated cats and goats differs significantly from that of the wild animals at the center of pandemic discussions, the underlying principle remains: contact between animals, humans, and food in crowded public spaces should be controlled, not made commonplace. Complicating the situation further is the fact that these animals are largely not unowned wild animals; they are pets of residents in the surrounding neighborhoods, left to roam unsupervised. This means that solutions cannot come solely from within the market. Coordination between market managers and the local Animal Husbandry Department is needed for systematic control of stray animals, as well as an educational campaign that reaches animal owners in the surrounding area, an intervention that is relatively low-cost but whose biosafety benefits can be felt far beyond the market walls.

As a policy implication rather than a finding directly supported by this study’s data, addressing OHS issues at Nanggalo Market sustainably requires more than physical improvements or management policy updates. It requires a governance mechanism rooted in the community and capable of long-term sustainability. The Occupational Health Post may offer a potentially suitable framework for this purpose, as a recognized community-based model within Indonesia’s public health system that reaches informal-sector workers who have traditionally fallen outside formal OHS protection [31]. Through the Community Health Center (Puskesmas) whose coverage area includes Nanggalo Market, the Occupational Health Post can be activated as a low-cost institutional platform for routine hazard monitoring, vendor health education, periodic health checks, and emergency response training. While its potential has long been recognized, the implementation of Occupational Health Posts in Indonesia’s traditional markets remains far from optimal, reflecting weak cross-sectoral coordination among health, trade, and market agencies. Activating the Occupational Health Post at Nanggalo Market is not simply adding a new service, but a real-world test of whether this existing model can work effectively in one of the most needed contexts, while also opening up opportunities for replication to thousands of other traditional markets in Indonesia and in developing countries with similar characteristics. We note that the feasibility and effectiveness of activating an Occupational Health Post specifically at Nanggalo Market were not directly evaluated within the scope of the present study; this recommendation is offered as a plausible, literature-informed direction for future intervention and implementation research, rather than as a tested finding of this study.

This study has several limitations that should be noted. Participants were recruited using purposive, non-probability sampling rather than random or stratified probability sampling; consequently, the sample cannot be assumed to be statistically representative of the full population of vendors, visitors, or management personnel at Nanggalo Market, and the elicitation frequencies reported in Section 3 should be read as indicators of relative hazard salience within this sample rather than as population-level prevalence estimates. The study was conducted at only one market location, so the findings are not necessarily broadly generalizable; comparative studies across traditional markets in other cities in Indonesia are needed in the future. Furthermore, while the elicitation method used was effective in capturing a comprehensive range of hazards, it did not yield quantitative data on the incidence or prevalence of injuries and health problems; linkage with health facility data from the local Community Health Center is recommended to strengthen the analysis in future research. Finally, the risk matrix approach relies primarily on subjective assessments of consequences and event probabilities, which can vary across raters; inter-rater reliability was not formally tested in this study, and this should be considered in the future development of similar methodologies. In addition, the elicitation guide and observation checklist used in this study were developed and internally reviewed by the research team but were not formally validated (e.g., through expert-panel content validation) or field pilot-tested prior to the main data collection (see Section 2.3); this may have affected the clarity, completeness, or consistency of hazard reporting across respondents. Finally, the study relied on self-reported hazard perceptions, structured observation, and interview data rather than objective exposure or accident/injury records (e.g., incident logs, medical records, or observed near-miss counts); consequently, the risk levels reported here reflect perceived and observed hazard characteristics rather than empirically measured exposure or incidence, and should be interpreted accordingly.

## 5. Conclusions

Conducted at a single traditional market using purposive sampling, this study’s findings should be interpreted as context-specific rather than broadly generalizable; with this caveat in mind, Nanggalo Market exhibits a complex work environment with far-from-adequate OHS risk controls for both traders and visitors. This study identified 10 hazards across six activity zones, seven of which were categorized as high risk according to the AS/NZS 4360:2004 matrix. The lack of technical and administrative controls for most of these high risks, including the absence of portable fire extinguishers and evacuation route signs, is a serious issue that requires an immediate response from market managers and local authorities. The multi-stakeholder elicitation approach employed in this study revealed significant differences in hazard perceptions among traders, visitors, and managers, highlighting the need for all stakeholders to engage in OHS data collection in traditional market environments. The proposed control recommendations are derived from the risk matrix and the ISO 45001:2018 hierarchy of controls, with technical (engineering) controls prioritized for hazards stemming from structural conditions; as a long-term measure, activating community-based Occupational Health Posts through primary health care facilities is tentatively proposed as a potentially affordable and sustainable OHS governance mechanism for this and similarly structured traditional markets in Indonesia; extrapolation to other developing-country contexts would require further site-specific evaluation. We emphasize that all control and governance recommendations in this manuscript, including the proposed hierarchy-of-controls interventions and the Occupational Health Post proposal, are derived from a descriptive risk assessment rather than from an intervention study, and their practical effectiveness at Nanggalo Market should be confirmed through future intervention or implementation research before being adopted as standard practice.

## Figures and Tables

**Table 1:** Primary potential hazards and associated risks identified by activity zone at Nanggalo Market, Padang.

Activity Zone	Primary Potential Hazard	Associated Risk
Traffic and Parking Area	Heavy traffic congestion on weekends and public holidays	Traffic accident/injury
	Absence of directional signage at market entry/exit	Congestion and vehicle collision
Buying and Selling Activities	Narrow circulation corridors between vendor stalls	Pickpocketing and physical fatigue
Culinary Activities	Absence of vendor zoning system	Visitor fatigue and disorientation
	Uncovered food displayed in open stalls	Foodborne illness (gastrointestinal)
Building, Tables, and Floor	Wet and slippery floors in the meat and fish section	Slip and fall injury
	Dark and inadequate toilet facilities	Fall and sanitation-related illness
Security and Stray Animals	Cats and goats entering the market area	Merchandise damage and zoonotic risk
Emergency Access	No evacuation route signage	Crowd crushing during emergency
	Absence of portable fire extinguishers	Uncontrolled fire

**Table 2:** Risk assessment matrix and control recommendations for identified hazards at Nanggalo Market (AS/NZS 4360:2004).

Activity	Hazard	Risk	C	L	Risk Level	Recommended Control
Traffic & Parking	Heavy weekend traffic	Traffic accident	3	B	High	Administrative: Coordinate with Transportation Agency for weekend traffic officers
Traffic & Parking	No entry/exit signage	Congestion	2	C	Moderate	Engineering: Install directional signage and parking guides
Buying & Selling	Narrow corridors	Pickpocketing/fatigue	3	B	High	Engineering: One-way traffic lanes; Administrative: Enforce stall display discipline
Buying & Selling	No vendor zoning	Visitor fatigue	3	C	High	Engineering: Redesign vendor zoning layout
Culinary	Uncovered food	Foodborne illness	2	C	Moderate	Elimination: Vector control with Health Department; Administrative: Separate raw/cooked food vendors
Building & Floor	Wet/slippery floor (fish/meat)	Slip and fall	3	B	High	Engineering: Replace floor surface with non-slip material; PPE: Safety boots for vendors
Building & Floor	Dark, inadequate toilets	Fall injury	2	B	High	Engineering: Improve toilet lighting and increase number of facilities
Security/Stray Animals	Cats and goats in market	Merchandise damage/zoonosis	1	B	Moderate	Elimination: Coordinate with Animal Husbandry Agency; Administrative: Educate pet owners
Emergency Access	No evacuation route signage	Crowd crushing	3	C	High	Engineering: Install evacuation route signage; Administrative: Regular emergency drills
Emergency Access	No fire extinguishers	Uncontrolled fire	4	D	High	Engineering: Provide fire extinguishers per zone; Administrative: fire extinguisher training for vendors

C = Consequence severity (1–5); L = Likelihood (A = rare to E = almost certain). Risk level determined by AS/NZS 4360:2004 matrix. High risk = immediate action required; Moderate risk = action to be scheduled.

## Data Availability

The original contributions presented in this study are included in the article/Supplementary Material. Further inquiries can be directed to the corresponding author.

## Supplementary Materials

The following supporting information can be downloaded at https://www.mdpi.com/article/10.3390/ijerph23070941/s1, Table S1: Full elicitation frequency data by respondent group and hazard item.
