# Supplementary material for: Hazard Identification and Risk Prioritization Among Vendors and Visitors of a Traditional Wet Market in Padang, West Sumatra, Indonesia
Source: Int J Environ Res Public Health. 2026 Jul 22;23(7):941. doi: 10.3390/ijerph23070941 (PMC13409861; doi:10.3390/ijerph23070941)
Supplement: Supplementary file 1 [file ijerph-23-00941-s001.zip › ijerph-4400359-supplementary.pdf]

Supplementary Materials

Table S1. Full Elicitation Frequency Data by Respondent Group and Hazard Item

*Elicitation was conducted with 45 participants: 20 vendors, 20 visitors, and 5 market management personnel. Participants were asked to freely identify hazards in each of six activity zones without prompting. Each cell reports the number of respondents (out of the subgroup total) who identified the hazard item. Shaded rows (yellow) indicate the primary potential hazard designated per zone (highest total frequency). * indicates the hazard selected as primary risk for the zone-level risk assessment.*

No	Hazard Item Identified by Respondents	Vendors (n=20)	Visitors (n=20)	Managers (n=5)	Total (n=45)
**Zone 1 — Traffic and Parking Area**					
**1**	**Heavy traffic congestion on weekends and public holidays**	**12**	**9**	**2**	**23**
2	Absence of directional signage at market entry/exit points	2	2	2	6
3	Loss or theft of merchandise/belongings in parking area	1	2	0	3
4	Traffic accidents reported during peak market days	2	1	0	3
5	Motorcycle theft incidents in parking area	2	0	0	2
6	General discomfort and inadequate parking facilities	3	0	1	4
7	Illegal parking obstructing traffic flow	0	2	0	2
Total reports — Zone 1		**22**	**16**	**5**	**43**
**Zone 2 — Buying and Selling Activities**					
**1**	**Narrow circulation corridors between vendor stalls**	**9**	**16**	**2**	**27**
2	Absence of a vendor zoning/categorization system	3	4	1	8
3	Poor environmental cleanliness in the trading area	1	1	0	2
4	Risk of falling due to uneven or slippery floor surfaces	1	0	0	1
5	Vendors not wearing face masks during transactions	0	2	0	2
Total reports — Zone 2		**14**	**23**	**3**	**40**
**Zone 3 — Culinary Activities**					
**1**	**Food items displayed uncovered, exposed to vectors and dust**	**3**	**3**	**0**	**6**
2	Inadequate cleanliness of food preparation and service areas	0	0	1	1
Total reports — Zone 3		**3**	**3**	**1**	**7**
**Zone 4 — Building Structure, Vendor Tables, and Floor Conditions**					
**1**	**Wet and slippery floors in fish and meat section**	**17**	**12**	**4**	**33**
2	Generally unclean floor conditions throughout the market	1	3	0	4
3	Leaking roof creating wet floor patches in multiple zones	1	2	0	3
4	Uneven floor surfaces creating trip hazards	2	3	2	7

5	Sloped or slippery staircase surfaces	3	2	1	6
6	Dark and inadequate toilet facilities	5	7	1	13
7	Insufficient waste bins at key locations in the market	1	0	0	1
8	Slippery staircase with no anti-slip treatment	1	0	0	1
9	Minor laceration injuries among fish and meat vendors	7	2	1	10
10	Clogged drainage channels with accumulated waste	1	2	0	3
Total reports — Zone 4		**39**	**33**	**9**	**81**
**Zone 5 — Security and Stray Animals**					
**1**	**Cats and goats entering the market area freely**	**4**	**9**	**2**	**15**
2	Overcrowding during peak hours	3	3	0	6
3	Stray animals consuming or contaminating merchandise	1	0	0	1
4	Lost goods or items mixed up between vendors/visitors	1	1	0	2
5	Presence of rats in market stalls and storage areas	3	0	0	3
6	Large numbers of cockroaches in food and storage areas	1	0	0	1
7	Theft of merchandise (reported by management)	0	0	2	2
8	Insufficient security personnel for market size	0	0	1	1
Total reports — Zone 5		**13**	**13**	**5**	**31**
**Zone 6 — Emergency Access**					
**1**	**No evacuation route signage displayed anywhere in the market**	**11**	**13**	**4**	**28**
2	Complete absence of portable fire extinguishers (APAR)	7	7	3	17
Total reports — Zone 6		**18**	**20**	**7**	**45**
**GRAND TOTAL — All Zones (38 hazard items)**		**109**	**108**	**30**	**247**

Notes: Yellow-shaded rows mark the primary hazard per zone (highest total frequency), designated as the primary potential hazard for risk assessment. **Red bold totals** indicate primary hazard total frequencies. n = number of respondents in each subgroup. All figures represent raw endorsement counts (not percentages).

Summary: Top 5 Most Frequently Reported Hazard Items (All Respondents)

Rank	Zone	Hazard Item	n	%
**1**	Zone 4	**Wet and slippery floors in fish and meat section**	**33**	73.3
**2**	Zone 6	No evacuation route signage displayed in the market	**28**	62.2
**3**	Zone 2	Narrow circulation corridors between vendor stalls	**27**	60.0
**4**	Zone 1	Heavy traffic congestion on weekends and public holidays	**23**	51.1
**5**	Zone 6	Complete absence of portable fire extinguishers (APAR)	**17**	37.8

% = percentage of total respondents (n = 45).
